# Identification and characterization of chemical components in the bioactive fractions of *Cynomorium coccineum* that possess anticancer activity

**DOI:** 10.7150/ijbs.38475

**Published:** 2020-01-01

**Authors:** Xiangmin Li, Mouna Sdiri, Juanjuan Peng, Yizhen Xie, Burton B Yang

**Affiliations:** 1 State Key Laboratory of Applied Microbiology Southern China, Guangdong Provincial Key Laboratory of Microbial Culture Collection and Application, Guangdong Institute of Microbiology, Guangdong Academy of Sciences, Guangzhou, 510070, China; 2 Sunnybrook Research Institute, Sunnybrook Health Sciences Centre, Toronto, M4N3M5, Canada; 3 Yuewei Edible Fungi Technology Co. Ltd., Guangzhou 510070, China; 4 Department of Laboratory Medicine and Pathobiology, University of Toronto, Toronto, M4N3M5, Canada

**Keywords:** *Cynomorium coccineum*, UPLC-Q-Orbitrap/MS, HepG2, LC3II, autophagy, cell apoptosis

## Abstract

*Cynomorium coccineum* has long been used as the health and medicinal plant known to induce cancer cell death. However, the bioactive compounds of *C. coccineum* and the underlying mechanism of their regulator in cell autophagy and cell apoptosis remain unexplored. In our previous study, we found that the ethanol extract had antitumor activity through inducing cancer cell death. In this study, by detecting the anti-tumor effect of sequence extracts from *Cynomorium coccineum*, the active constituents were collected in solvent ethyl acetate. A strategy based on ultra-performance liquid chromatography coupled with hybrid quadrupole-orbitrap mass spectrometry (UPLC-Q-Orbitrap/MS) was first utilized to analyze the chemical constituents of active fraction (ethyl acetate fraction, CS3). A total of 29 compounds including 8 triterpenoids, 6 flavonoids, 4 fatty acids, 8 phenolic acids, 1 anthraquinones, 1 nucleoside and 1 sterol were detected and identified or tentatively identified for the first time in *Cynomorium coccineum*. We found that CS3 induces cancer cell death accompanied with a great number of vacuoles in the cytoplasm. CS3-induced autophagosome formation was found and confirmed by electron microscopy and the high expression levels of microtubule-associated protein-1 light chain 3-II (LC3II), a marker protein of autophagy. We additionally demonstrated that CS3 activated and increased the pro-apoptotic mitochondrial proteins, BNIP3 and BNIP3L, in mRNA and protein levels. The constituents of CS3 down-regulated anti-apoptotic BCL2, and then releases autophagic protein Beclin-1. These finding for the first time systematically not only explore and identify the active constituents of CS3 in *Cynomorium coccineum*, but also examined the mechanism associated with CS3-induced cell death via cell autophagy. This active component may serve as a potential source to obtain new autophagy inducer and anti-cancer compounds for hepatocellular carcinoma.

## Introduction

Cynomoriaceae is a well-known family of medicinal and parasitic plants, which contains the sole genus *Cynomorium* including two species* Cynomorium coccineum* L. and *Cynomorium songaricum* Rupr 1,2.* Cynomorium coccineum* is found in Northern Africa and in the Mediterranean region, where it has a long history of being regarded as health food and folk medicine for prevention and treatment of various diseases 2,3. In recent years, the ethanol and aqueous extracts of* Cynomorium coccineum* have been studied extensively for their effects on anti-cancer, anti-diabetic, anti-oxidative, improving immunity and HIV-1-protease inhibition with *in vivo* and *in vitro* models 1,3-6. To date, the literatures for the chemical compositions of *Cynomorium coccineum* only reported approximately 14 fatty acids 2, which have made the investigation of the chemical components of *Cynomorium coccineum* extremely difficult.

In this study, we aimed to identify the chemical constituents and investigate the underlying mechanisms mediating the anti-cancer activity from the active fraction of *Cynomorium coccineum*. The UPLC coupled with Q-Orbitrap/MS method has the characteristics of high efficient chromatographic separations, mass resolution and multistage MS fragment ions, which has spread in the field of medicinal herbs analysis 7. Benefiting from the advantage of particle columns (<2µm), UPLC provides highly efficient and fast separation of compounds. Compared with the other MS instruments, Q-Orbitrap/ MS showed a significantly high-resolution and accurate-mass. In a word, UPLC-Q- Orbitrap/MS is a more effective and accurate tool for high performance separation and identification of the unknown secondary metabolites from natural resources. Thus, UPLC couple with Q-Orbitrap/MS was utilized to analyze and identify the chemical constituents in bioactive fraction (ethyl acetate fraction, CS3) from *Cynomorium coccineum*. A total of 29 compounds of interest were detected and identified or tentatively identified, which were mainly attributed triterpenoids and flavonoids. We further investigated the compositions of these active components. Mitochondria-associated cell death protein BNIP3 and BNIP3L that play important roles in the autophagic and apoptotic cell death are the target molecules in our study.

## Materials and Methods

### Materials

Acetonitrile (ACN) and methanol (MeOH) for UPLC analysis were supplied by Thermo fisher (USA). HPLC grade formic acid (FA) was purchased from Sigma-Aldrich (USA). All other analytical grade chemicals were obtained from Guangzhou Chemical Reagent Factory (Guangzhou, China). Deionized water was purified by the Millipore-Q water purification system (USA). *Cynomorium coccineum* were collected in the Sahel region of Northeastern Tunisia. The monoclonal and polyclonal antibodies against LC3B, Bcl-2, BNIP3, BNIP3L (NIX) were purchased from Cell Signaling Technology. Alexa Fluor 555, phalloidin (Alexa Fluor 488), and DAPI purchased from Thermo fisher. Horseradish peroxidase-conjugated goat anti-mouse IgG and goat anti-rabbit IgG were obtained from Cell Signaling Technology. RNA extract kits, RNA RT and polymerase chain reaction (PCR) kits were obtained from Thermo Fisher. Immunoblotting was performed using the Enhanced Chemiluminescence (ECL) western blot detection kit (Millipore). 3-methyladenine and β-actin were purchased from sigma.

### Preparation of *Cynomorium coccineum* extract

*Cynomorium coccineum* powder (1.25 kg) was soaked in 95% ethanol at a ratio of 1:15 (*w*/*v*) and extracted by refluxing three times at 80°C, 2 h each. The extracted solution was filtered to get supernatants followed by precipitation to concentrate the bioactive components (CS1, 130 g). Furthermore, the precipitates were extracted with 75% ethanol by refluxing three times at 80°C, 2 h each. The extracted solution was filtered and concentrated (CS2, 360 g). CS2 was then fractionated by ethyl acetate, water-saturated butanol, and water alone stepwise. After evaporation of the collection, ethyl acetate fraction (CS3, 12 g), water-saturated butanol fraction (CS4, 125 g), and water fraction (CS5, 220 g) were obtained (for detail procedure see Fig. S1).

### Liquid chromatography and mass spectrometry analysis of CS3

In this study we used an Ultimate 3000 UPLC tandem Q-Exactive/MS spectrometer (Thermo Fisher, CA, USA) equipped with a heated electrospray ionization (HESI) probe for purification of the anticancer ingredient. Samples were separated on Hypersil Glod C18 column (1.9 μm, 50 × 2.1 mm, Thermo Fisher, USA) at the flow rate of 0.3 mL/min and the column temperature was maintained at 30°C. The mobile solvent system consisted of 0.1% aqueous formic acid (v/v) (A) and methanol (B). The samples were eluted with the following linear gradients: 2%~5% B at 0~2 min;5%~95% B at 2 min~13 min; 95%~5% B at 13 min~15 min; 2% B at 15 min~18 min. Injection volume was 3 μL.

Detection was performed using a Q Exactive Focus Hybrid Quadrupole-Orbitrap mass spectrometer in both positive and negative ionization modes. The optimal analysis conditions were set as follows: ion source, heated electrospray ionization probe; source temperature: 350°C; capillary temperature: 320°C; sheath gas: 45 arb; auxiliary gas: 8arb; mass collecting range: m/z 100~1500. The full scan and dd-MS^2^ spectra were collected at the resolutions of 70000 and 17500, respectively.

MS data were acquired in both negative and positive ion modes and processed for compounds identification using a combination of Xcalibur 4.1 and compound Discoverer 3.0 (Thermo Fisher, CA, USA). Predicting the possible elemental compositions of the potential compositions in *Cynomorium coccineum*, the types and numbers of expected atoms were set as follows: carbons≤50, hydrogens≤100, oxygens≤50, nitrogen≤10. The mass accuracy error threshold was set at 5 ppm.

### Cell proliferation assay

Cell proliferation assay was performed to evaluate the anticancer effect of different fractions of *Cynomorium coccineum* by trypan blue staining as described 8-10. Human hepatocellular carcinoma cells HepG2 (from ATCC), human breast cancer cells MDA-MB-231 (from ATCC), MCF-7 (from ATCC), and human prostate cancer cells LNCaP (from ATCC) were used in the study. The cells (1x10^5^ cells/mL, 0.5 mL/well) were seeded into 24-well plates and cultured in DMEM/RPMI 1640 containing 10% FBS with 100 U/mL penicillin/streptomycin at 37°C in an incubator containing 5% CO_2_. Four hours after cell inoculation, the fractions of *Cynomorium coccineum* extract (CS1, CS2, CS3, CS4, CS5) were added to the cultures at the different concentrations and incubated for 48 h or up to for 72 h. We also tested the effects of the extract on normal human breast cell lines MCF-10A (from ATCC). Cell viability was analyzed by trypan blue staining. Each experiment was repeated for at least three times for statistical analysis.

### Flow cytometer assay

Cell apoptosis was analyzed with flow cytometry as described 11,12 . HepG2 cells were treated with CS3 (25 and 75μg/ml), washed, trypsinized (EDTA-free), and harvested (keeping the floating cells together). The cells were washed with cold PBS, resuspend in binding buffer, and incubated with PE Annexin V and 7-AAD following the instructions of PE Annexin V apoptosis detection kit (BD, USA). The cells were analyzed by flow cytometry (FACS CantoII, BD, USA). Percentages of the cells with PE Annexin V positive staining were considered as apoptotic, whereas 7-AAD-positive staining was considered to be necrotic.

### Fluorescence microscopy

HepG2 cells were transfected with GFP-LC3 plasmid. After 24 h, the cells were treated with CS3. Fluorescence of GFP-LC3-transfected cells was examined, and the images were generated via EVOS FL imaging system (Thermo Fisher, USA).

Immune-fluorescent assay was performed as described to further examine subcellular localization of LC3B in the HepG2 cells treated with or without CS3 13,14. In brief, HepG2 cells were also seeded on chamber slides (Thermo Fisher, USA) and treated with or without CS3 (20 and 40 μg/ml, respectively) for 24 h. The cells were washed three times with PBS, fixed with 4% paraformaldehyde for 15 min, and blocked with 10% goat serum in PBS. The cells were incubated with anti-LC3 antibody at 4°C overnight. After rinse three times with PBS, the slides were incubated with Alexa Fluor 555 secondary antibody at room temperature for 2 h in a dark container. The slides were rinsed with PBS and incubated with phalloidin (Alexa Fluor 488) for F-actin staining at room temperature for 2 h in a dark container. The slides were then rinsed with PBS and incubated with DAPI for nuclear staining in the dark for 30 min. After being washed with PBS and mounted with mounting medium. The slides were subjected to image examination via EVOS FL imaging system (Thermo Fisher, USA).

### Electron microscopy

Briefly, HepG2 cells treated with CS3 were washed three times with PBS, trypsinized, and harvested by centrifugation. The cell pellets were fixed with 2.5% glutaraldehyde overnight at 4°C, post fixed with 1% osmic acid at the room temperature for 1 h and dehydrated using graded ethanol. The dehydrated pellets were rinsed with acetone and then embedded in resin for sectioning. Thin sections were observed and photographed under a transmission electron microscope (HT7700, Japan).

### RNA extraction and RT-PCR

This was performed as described previously 9,15. In brief, HepG2 cells treated with CS3 were harvested, and total RNA was extracted using TRIzol reagent according to the manufacturer's instruction (Invitrogen, 15596018). The total RNA concentrations were measured by Nanodrop One (Thermo Fisher, USA). The RNA was subject to reverse transcription using SuperScript Ⅳ VILO Master Mix (Invitrogen, USA) to obtain cDNAs. Real-time PCR was performed with a PowerUp SYBR Green master mix (ABI). The primers amplifying human U6 were used as an internal control for real-time PCR.

### Western blot analysis

HepG2 cells with CS3 treatment were lysed in lysis buffer containing protease inhibitors and the protein samples were subject to western blotting as described 16.

### Statistical Analysis

Results were analyzed by a Student's t-test. The levels of significance were set at *P < 0.05 and **P < 0.01.

## Results

### Screening active components from *Cynomorium coccineum*

**Fig 1:**
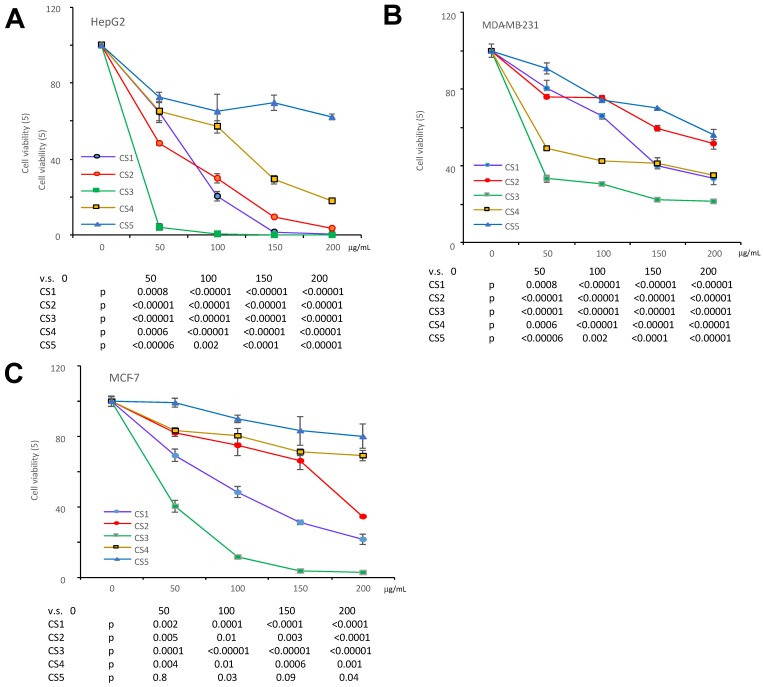
The different fractions of *Cynomorium coccineum* extract on cancer cell proliferation. The *Cynomorium coccineum* extract of CS1, CS2, CS3, CS4, CS5 were used to treat HepG2 cells (A), MDA-MB-231 cells (B) and MCF-7 cells (C) for 48 hours in gradient concentrations as indicated. Cell viability was determined. Each experiment was repeated three times. Data represent the mean ± SD of three independent experiments.

We have previously reported that the ethanol extract of *Cynomorium coccineum* inhibited cancer cell growth in vivo and vitro 3. To examine the biological activities of *Cynomorium coccineum* in detail, we purified the anticancer components of the extract. During product purification, we monitored its activities by incubating the fractions with cancer cell cultures. We prepared 100% ethanol extract (CS1), 75% ethanol extract (CS2) and solvent-partitioned fractions (CS3-CS5) from the dried fruiting bodies of *Cynomorium coccineum*. The products were used to treat three different human cancer cell lines (HepG2, MDA-MB-231, and MCF-7). CS1, CS2 and CS3 were found to possess a potent effect on inducing death of all used cancer cells (Fig. 1). As showed in the figure, CS3 (ethyl acetate fraction), which was purified from the 75% ethanol extract, displayed clearly the strongest inhibitory effect on cancer cells at the same concentrations used (Fig. 1). The results indicated the most bioactive compounds of ethanol extract against cancer cells could be enriched in the CS3 fraction.

### UPLC-Q-Orbitrap/MS analysis of CS3

In recent years, benefiting from the advantage of particle columns (<2 µm) and Q-Orbitrap/MS that own significant high-resolutions and accurate-mass, UPLC- Q-Orbitrap/MS was used increasingly for the identification and characterization of components in the field of medicinal herbs 17. *Cynomorium coccineum* and *Cynomorium songaricum* belong to genus *Cynomorium*, in which the types of chemical components were deduced to own the similar structures. All the reported compounds in *Cynomorium songaricum* were consulted and attributed into seven types on the chemical structures: triterpenes, steroids, lignans, flavonoids and other phenolics, n-butyl-fructoside, and others, the molecular weight of which was in the 100-1500 Da 1,18,19. Therefore, UPLC-Q-Orbitrap/MS was selected to analyze the chemical constituents in the CS3 of the 75% ethanol extracts from *Cynomorium coccineum*.

**Fig 2:**
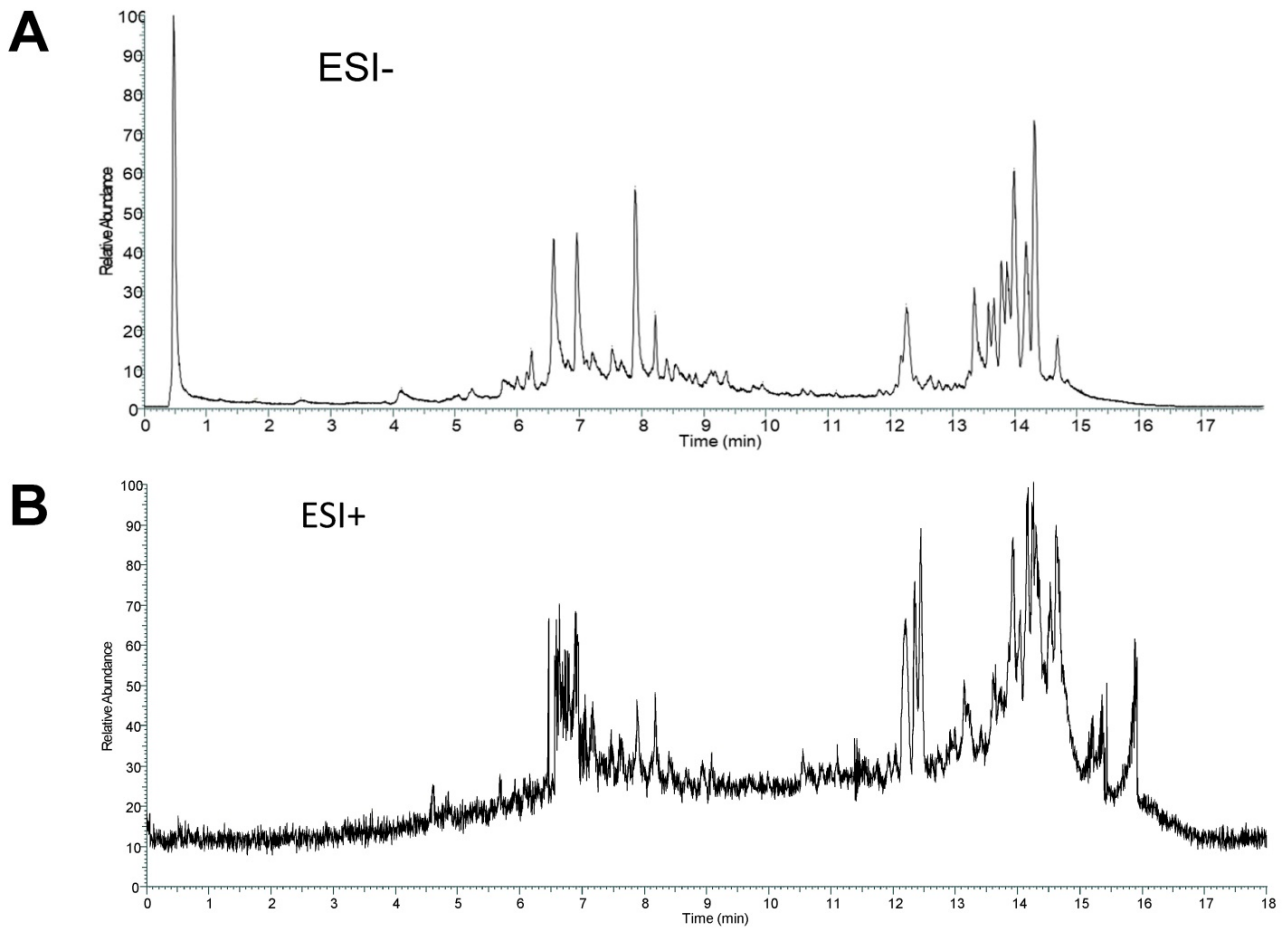
** ESI modes**. Total ion current chromatograms (TIC) of CS3 from *Cynomorium coccineum* in negative (A) and positive (B) ESI modes are shown.

The total ion current chromatogram (TIC) in negative and positive ESI modes was shown in **Figure 2**. The compounds in CS3 were discovered and identified according to molecular weight calculation, pyrolysis rule, characteristic fragment ion information and related literatures as follows: to obtain the molecular weight and formulas, a throughput search were performed by matching in the online database (ChemSpider, mzCloud); then, a manual search and match were carried out to identify compounds belonging to structural types by exact MS/MS data and fragment information. Finally, a total of 29 compounds were identified or tentatively identified in *Cynomorium coccineum* for the first time, including 8 triterpenoids, 6 flavonoids, 4 fatty acids, 8 phenolic acids, 1 anthraquinones, 1 nucleoside and 1 sterol (Table 1). Furthermore, it was found that the chromatography retention time of different types of chemical constituents in CS3 eluted with gradient methanol and aqueous 0.1% FA in UPLC were as the following order (from 0-18 min): phenolic acids < flavonoids < triterpenoids < fatty acids. In addition, we also found that most compounds showed stronger response in the negative ion mode (Fig. 2A) than positive ion mode (Fig. 2B). It has been reported that those compounds with phenolic hydroxyl group showed stronger response in the negative ion mode 20.Thus, the MS data for ergosterol peroxide, oleanolic acid and Betulin were analyzed in positive, others were in negative mode.

### CS3 inhibits cell proliferation and induces cell apoptosis

**Fig 3:**
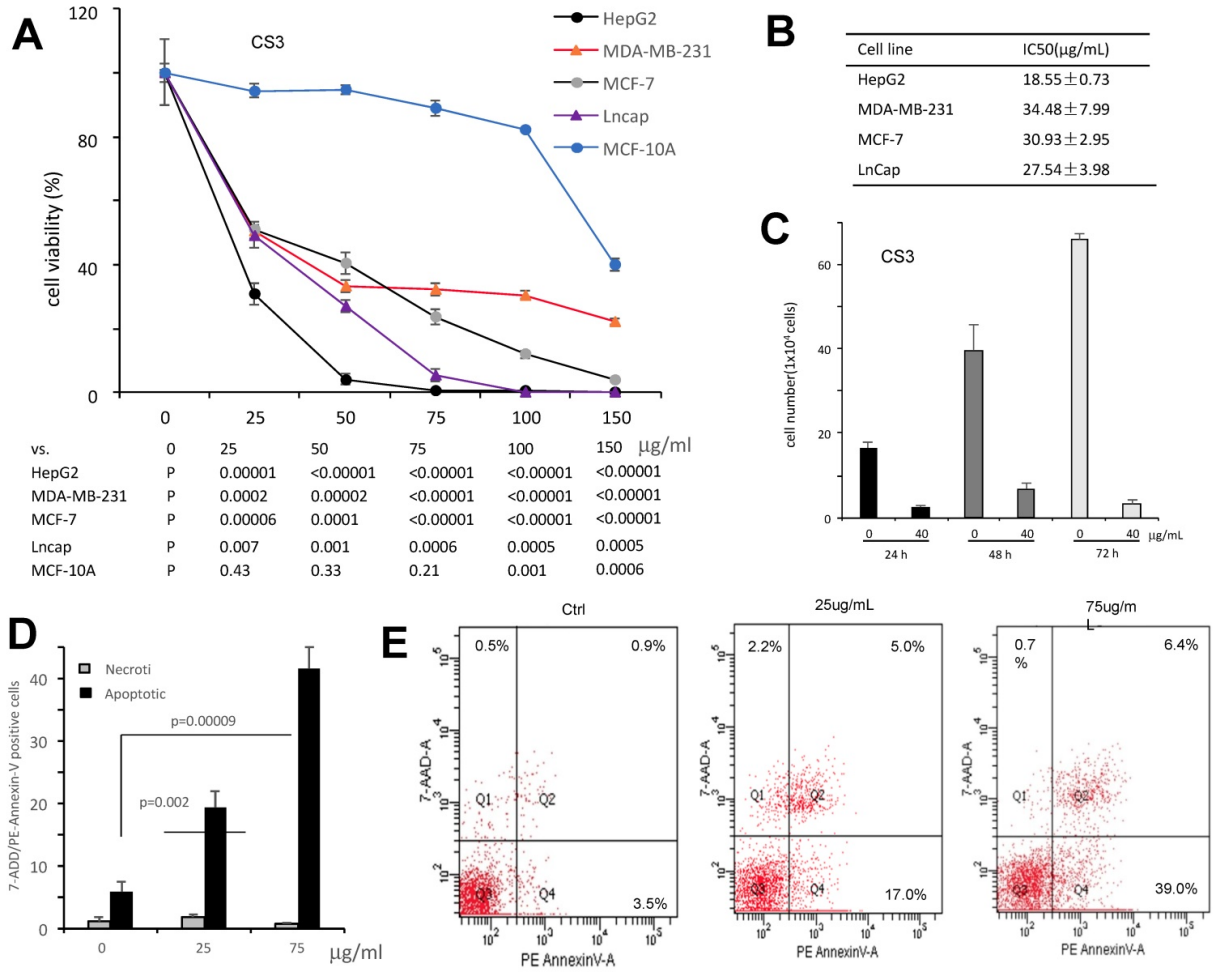
** The bioactive component CS3 inhibits cancer cells proliferation and induces cell apoptosis.** (A) Cells of HepG2, MDA-MB-231, MCF-7, and LNCaP were treated with CS3 for 48 hours by gradient concentrations. The normal breast epithelial cell line MCF-10A was used as a control. Cell viability was determined by trypan blue staining. (B) IC50 was calculated for each cell lines treated with CS3 for 48 h. (C) Hep G2 cells were treated with CS3 for up to 72h. Cell viability was determined by trypan blue assay. (** p<0.001, n=4). (D) The apoptosis and necrosis of Hep G2 cells induced with CS3 (25μg/mL, 75μg/mL, 24h) were determined by flow cytometer. Apoptotic: PE - Annexin V-positive; Necrotic: 7-AAD-positive. The data are presented as mean ± SD from three independent experiments. (** p<0.001, n=4). (E) Typical patterns of apoptosis and necrosis of Hep G2 cells are shown.

It has been shown that triterpenoids and sterols possess antitumor effect 21-23. We thus examined the effect of CS3 on inhibiting cancer cell proliferation with four different cancer cell types including HepG2, MDA-MB-231, MCF-7 and LnCap. The results showed that CS3 reduced cell viability in a dose-dependent manner (Fig. 3A). In the half maximal inhibitory concentration (IC50) measurements, we found that the human hepatocellular carcinoma cell line HepG2 was most sensitive to CS3 treatment (IC50: 18.5±0.73 µg/ml, Fig. 3B). Thus, we used the human hepatocellular carcinoma cell line HepG2 for further study. Figure 3C showed that proliferation of HepG2 cells was inhibited in a time-dependent manner when the cells were treated with CS3. Importantly, CS3 showed little effect on the viability and morphology of MCF-10A mammary epithelial cells, up to 150 µg/ml (Fig. 3A), suggesting specific effect of CS3 on cancer cells.

To verify whether CS3 decreased the viability of human hepatocellular carcinoma cells HepG2 through cell apoptosis, we used flow cytometer assay to detect the percentage of cells apoptosis. Treated with CS3 for 24 h, HepG2 cells showed increased percentage of apoptosis in a dose-dependent manner, compared with the control (p<0.05 at 25 µg/ml, p<0.01 at 75 µg/ml, Fig. 3D). Typical patterns of apoptosis and necrosis of Hep G2 cells are provided in Figure 3E.

### CS3 enhances autophagy in HepG2 cells

**Fig 4:**
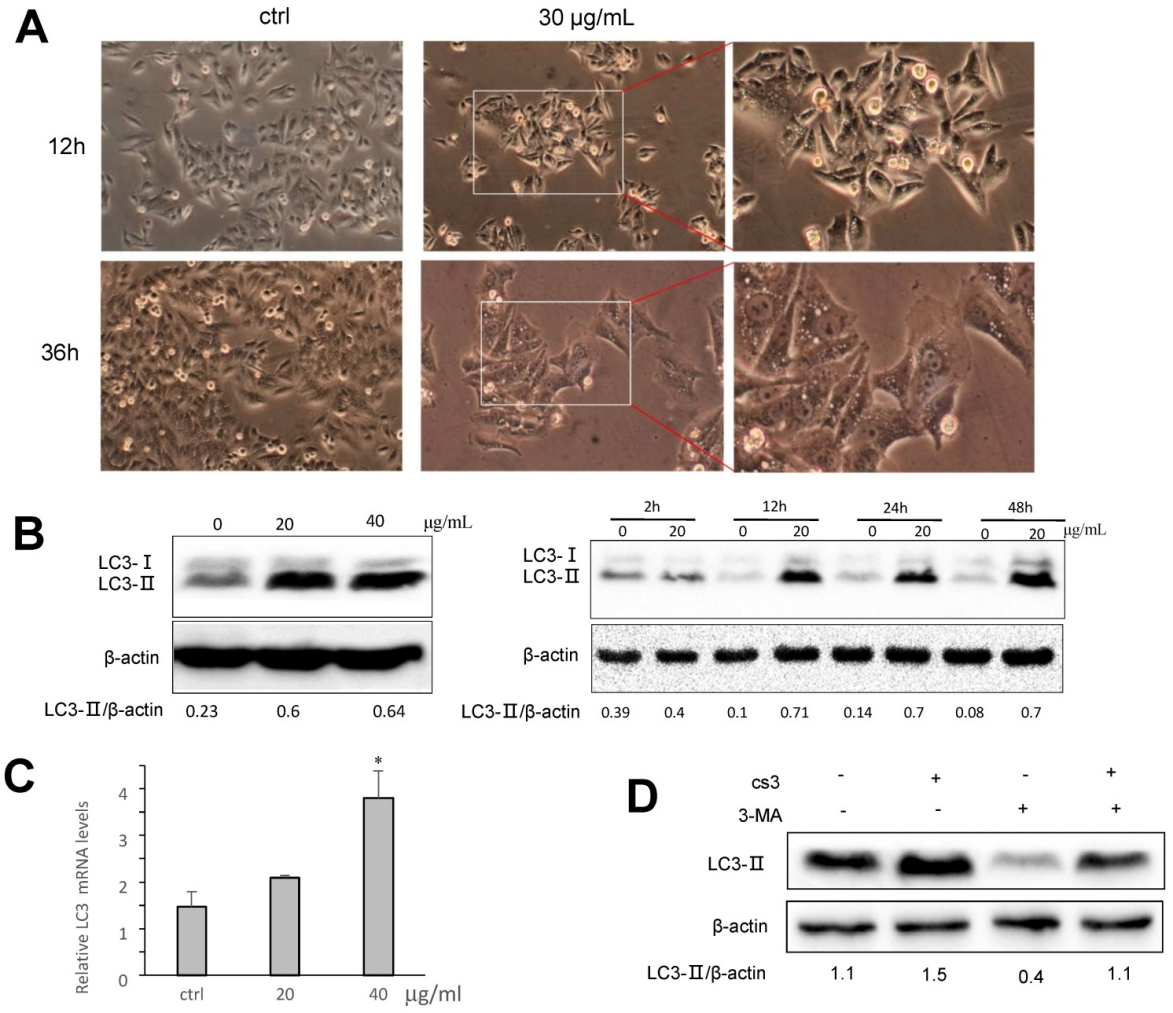
CS3 induces accumulation of LC3B. (A) HepG2 cell morphology was observed on CS3-treated (30μg/mL, 12h, 36h) under a microscope. (B) Left, HepG2 cells were treated with CS3 (0-40 μg/mL) for 24 h. Increased concentrations of CS3 promoted LC3B formation. Right, HepG2 cells were treated with CS3 (40 μg/mL) for up to 48 h. Longer treatment with CS3 generated higher levels of LC3B. Densitometry was performed for quantification and the ratios of LC3-II to β-actin are presented below the blots. (C) HepG2 cells were treated with CS3 (0-40 μg/mL) for 24 h. Cells were harvested followed by real-time PCR measurement of LC3 mRNA. (* p<0.05, n=4). (D) HepG2 cells were treated with CS3 (40 μg/mL) for 24 h in the presence or absence of 3-MA (2mM). Cell lysate were prepared and subject to Western blotting detected by anti-LC3-II antibody. Densitometry was performed for quantification and the ratios of LC3-II to β-actin are presented below the blots.

Many obvious vacuoles in cytoplasm were detected in the CS3-treated HepG2 cells (Fig. 4A). These vacuoles looked like autophagosome. We examined whether CS3 treatment could induce autophagosome formation, indication of cell autophagy. Microtubule-associated protein-1 light chain 3 (LC3) has three isoforms in human cells (LC3A, LC3B and LC3C) during autophagy 12,24,25. Two forms of LC3, named LC3-Ⅰ and LC3-II, were produced post-translational modifications in the cells 24,25. During autophagy, LC3-Ⅰ is converted to an autophagosome-associating form LC3-II 26,27. Since the LC3-II/actin ratio is an accurate indicator of autophagy, we analyzed the protein expression of LC3-II and found that the ratio of LC3-II to actin was significantly increased in CS3-treated cells, compared with the control cells (Fig. 4B). The effect of CS3 was concentration-dependent and time-dependent (Fig. 4B). The mRNA levels of LC3 was also measured by real-time PCR. The data showed that CS3 treatment increased the mRNA levels of LC3 compared to the control (Fig. 4C). To explore the possible association of CS3-induced cell autophagy, the autophagy inhibitor 3-Methyladenine (3-MA) was evaluated in the follow-up experiments. Pre-treatment of HepG2 cells with the inhibitor 3-MA prevented partially CS3-induced LC3II formation (Fig. 4D).

**Fig 5:**
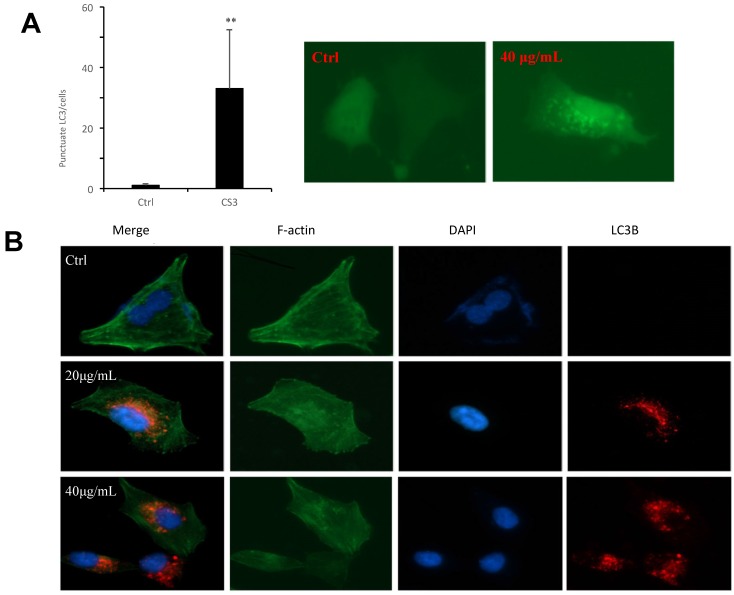
Formation of autophagosome examined by immunofluorescence. (A) HepG2 cells were transfected with plasmid expressing GFP-LC3. After 24 h, the cells were treated with DMSO (ctrl) or CS3 (40 μg/mL) for 36 h. The number of punctates GFP-LC3 in each cell was counted and at least 50 cells were included for each group. (** p<0.001, n=4). (B) Immunofluorescence using the antibody of LC3B were performed to stain HepG2 cells following treatment with CS3 (20, 40 μg/mL) for 24 h. Red dots were LC3B. The numbers of the punctate LC3B in each cell were counted, and at least 50 cells were included for each group.

To further examine autophagosome formation in the cells treated with CS3, HepG2 cells were transfected with a green fluorescent protein (GFP) and LC3 fusion protein and observed by EVOS FL imaging system. We found that CS3 increased the number of GFP-LC3 punctate in the transfected cells, while GFP-LC3 remained diffuse in the control cells (Fig. 5A, p<0.01). Meantime, we also examined the expression of LC3II in the HepG2 cells treated with or without CS3 by immuno-fluorescent assay. It showed that LC3II expression was clearly increased in the HepG2 cells after CS3 treatment as compared to the control (Fig. 5B, p<0.01).

**Fig 6:**
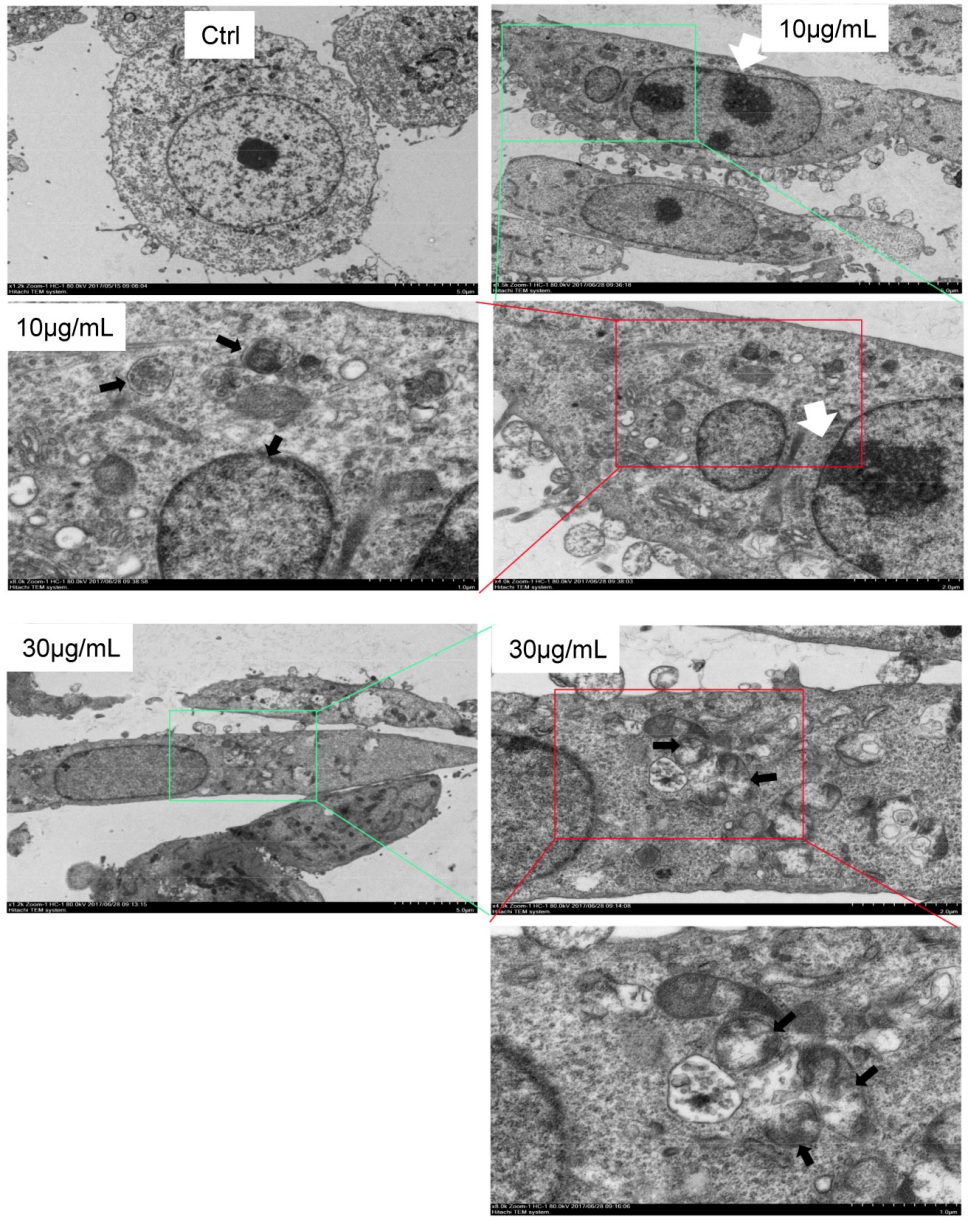
Formation of autophagosome examined by electron microscope. Electron microscope was performed on Ctrl and CS3-treated (10, 30 μg/mL, 12 h) HepG2 cells. Black arrowheads, a vacuole with double membrane structure and organelle; white arrowheads, the condensation and margination of chromatin on the nuclear membrane.

Finally, we used electron microscopy to directly detect autophagosome, which is one of the most convincing approaches in autophagy study. The cells were treated with CS3 followed by sample preparation for electron microscopic examination of autophagosome. The CS3-treated cells showed many membrane vacuoles accumulated in the cytoplasm of the CS3-treated HepG2 cells, compared with control cells. We could clearly detect the double membrane structures of autophagosome vacuoles (black arrowhead, high magnification) in the CS3-treated HepG2 cells (Fig. 6). In addition, we detected the condensation and margination of chromatin on the nuclear membrane (Fig. 6, white arrowhead) that were characteristics of cell apoptosis. These results indicated that CS3 induced autophagy and apoptosis in the same cells.

### CS3 regulates cell autophagic proteins and cell apoptotic proteins

**Fig 7:**
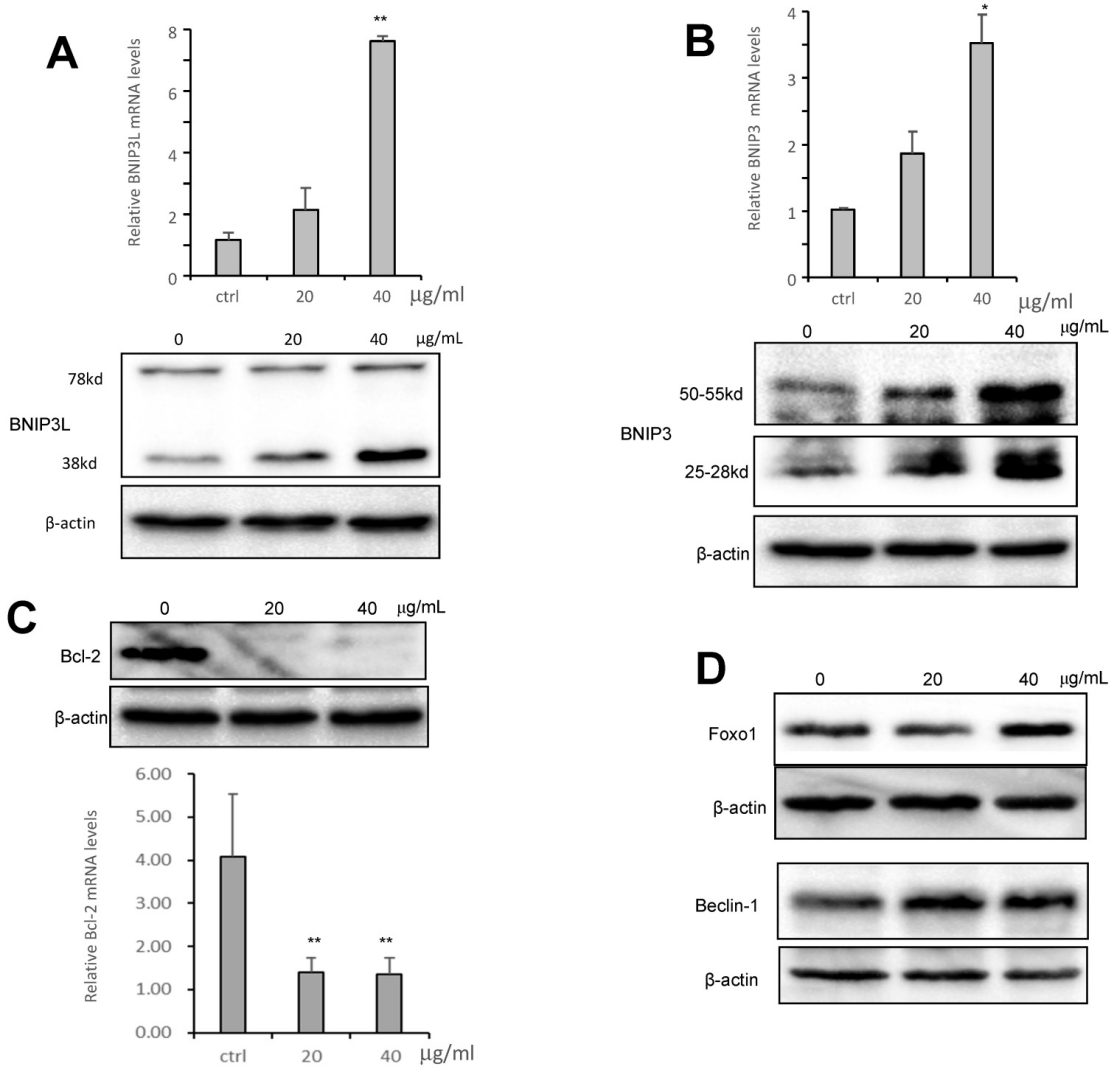
CS3 modulates mRNA and protein expression of BNIP3, BNIP3L and Bcl2. (A) HepG2 cells were treated with CS3 (0-40 μg/mL) for 24 h. Upper, cells lysate was prepared and analyzed by western blotting probed with anti-BNIP3L antibody. Lower, cells were lysis, and total RNAs were extracted followed by measurement of BNIP3L mRNA by real-time PCR. (** p=0.001, n=4). (B) HepG2 cells were treated with CS3 (0-40 μg/mL) for 24 h. Upper, cells lysate was prepared and analyzed by western blotting probed with anti-BNIP3 antibody. Lower, cells were lysis, and total RNAs were extracted followed by measurement of BNIP3 mRNA by real-time PCR. (** p<0.05, n=4). (C) HepG2 cells were treated with CS3 (0-40 μg/mL) for 2 4h. Upper, cells lysate was prepared and analyzed by western blotting probed with anti-Bcl-2 antibody. Lower, cells were lysis, and total RNAs were extracted followed by measurement of Bcl-2 mRNA by real-time PCR. (** p=0.001, n=4). (D) HepG2 cells were treated with CS3 (0-40 μg/mL) for 24 h. Cells lysate was prepared and analyzed by western blotting probed with antibodies against Foxo1 and Beclin-1.

To examine the molecular mechanism associated with CS3-induced autophagy, we tested expression of key molecules in the autophagy pathway. We tested whether CS3 induced cell autophagy and cell apoptosis through regulating expression of the association proteins. BNIP3 (Bcl-2/ adenovirus E1B-19kDa interacting protein 3) and BNIP3L (Bcl-2/adenovirus E1B 19-kDa protein-interacting protein 3-like, NIX) are proteins with homology to Bcl-2 in the BH3 domain, which induce both cell death and autophagy 28,29. We explored the potential roles of BNIP3 and BNIP3L in mediating CS3's effect on inducing cell death and autophagy. After treatment of HepG2 cells with CS3 for 24 h, RT-PCR analysis showed that the mRNA expression of BNIP3 and BNIP3L were significantly up-regulated in a concentration-dependent manner as compared with the control (Fig. 7A, 7B, upper). Western blot analysis also displayed a concentration-dependent effect of CS3 on BNIP3 and BNIP3L protein levels (Fig. 7A, 7B, lower).

BNIP3L and BNIP3, when localized to mitochondria, can bind Bcl-xL and Bcl-2, which induces cell death 28,30. We detected expression of Bcl-2 and Bcl-xL. RT-PCR and Western blot showed that the mRNA and protein levels of Bcl-2 were significantly down-regulated as compared with the control (Fig. 7C), but the expression of Bcl-xL was not affected Fig. S2. In addition, we also examined the expression of other proteins that are associated with cancer cell apoptosis and autophagy including Foxo1 and Beclin-1. HepG2 treated with CS3 greatly increased expression of Foxo1 and Beclin-1 (Fig. 7D), confirming the induction of CS3 in cancer cell apoptosis and autophagy.

## Discussion

*Cynomorium coccineum* L. and *Cynomorium songaricum* Rupr. are the only two species in the genus* Cynomorium*, which have been extensively investigated for their pharmacological effects. Over the past decades, it has been demonstrated that the main bioactive constituents of *Cynomorium songaricum* are polysaccharides, triterpenes, steroids, lignans, flavonoids and other phenolics, n-butyl-fructoside, and others. However, the only reported constituents of *Cynomorium coccineum* are fatty acids for antioxidant and scarcely studied 30. Our previous study clearly showed that *Cynomorium coccineum* possesses strong anticancer effects 3. We thus reasoned that some other compounds must be playing key role in pharmacological effects of *Cynomorium coccineum* in the induction of cancer cell death. In this study, after a series of fractionation and active screening, we found that ethyl acetate fraction (CS3) was the major compounds in inducing cancer cell death. According to ethanol and ethyl acetate solvent polarity, the types of components in CS3 were mainly small molecules. Thus, UPLC couple with Q-Orbitrap/MS was utilized to analyze and identify the chemical constituents in CS3 purified from *Cynomorium coccineum*. Based on the strategy of characteristic product ions and fragmentation rules of various types of compounds, a total of 29 compounds of interest were detected and identified or tentatively identified in CS3, which were mainly attributed triterpenoids and flavonoids. To date, over 50 compounds have been isolated and identified from *Cynomorium* plants, but only two flavonoid glycosides, cyanidin 3-O-glucoside and cyanidin 3-O-(6-O-rhamnosylglucoside), have been isolated from *Cynomorium coccineum* 19,31. Because the lack of reference compounds from *Cynomorium coccineum*, the compounds were not further identified and characterized. Despite this, this is the first study to systematically analyze the chemical constituent profile of *Cynomorium coccineum* by UPLC-Q-Orbitrap/MS, and the results will provide essential guide to purify active compounds and explore antitumor molecular mechanisms. Since *Cynomorium songaricum* is the other species of the genus, our study will also provide useful information for those who are studying the anticancer activity of* Cynomorium songaricum*.

It has been reported that the triterpenoids and flavonoids compounds induce cancer cell death and autophagy 32,33. Our analysis of chemical constituents provided strong evidence showing that CS3 inhibited cancer cell proliferation and induced cell death in dose- and time-dependent manners. More importantly, we found that CS3 induced cell autophagy. The formation of autophagosome was identified by electron microscopy in CS3-treated HepG2 cells. It has been known that LC3 is a specific marker to detect autophagosome formation 34. The CS3-induced autophagy was further confirmed by immuno-fluorescence and immunoblotting for lipidation of LC3 and was prevented by autophagy inhibitor (3-MA). The electron microscopic photographs also showed the presence of the autophagic vacuoles and the characteristics of apoptosis. Additionally, we found that mitochondria-associated cell death protein BNIP3 and BNIP3L played an important role in CS3-induced cell autophagy and cell death. BNIP3 and BNIP3L, the pro-apoptotic BH-3-only mitochondrial proteins, induce both cell death and autophagy, which are suppressed by Bcl-2 28. The antiapoptotic function of Bcl-2 has been described and reviewed numerous times 35,36. Our results showed that Bcl-2 were clearly down-regulated in CS3-treatment HepG2 cells. We also detected that Beclin-1 were up-regulated in HepG2 cells after CS3 treatment. It has reported BNIP3L can stimulate the induction of autophagy by dissociating the Bcl-2-Beclin-1 complex through competitively binding to Bcl-2 by means of its BH3 domain 37. BNIP3 has also been described in autophagic cell death 38. Thus, we conclude that the CS3-induced cell autophagy, once overactivated, lead to cancer cell death.

In summary, we investigated the anti-cancer activity of *Cynomorium coccineum.* The constituents of anti-cancer activity (CS3) were obtained, which were monitored by their anticancer activity and purified in the 75% ethanol extract. A rapid and efficient UPLC-Q-Orbitrap/MS method was first established for bioactive components in *Cynomorium coccineum*. A total of 29 compounds of interest were detected and identified or tentatively identified on systematic fragment ion strategy. The MS^n^ fragmentation patterns of the compounds were also explored and summarized in positive or negative ion modes. We have thus demonstrated that the composition of CS3 can activate and enhance cellular autophagic process to triggering apoptosis. Mitochondria-associated cell death protein BNIP3 and BNIP3L play an important role in the autophagic and apoptotic cell death. These finding for the first time systematically explore and identify the active constituents of CS3 in *Cynomorium coccineum*. Our study also suggests a novel concept to the active constituents of CS3-induced cell death pathways. Further work could obtain and identify monomeric compounds to obtain autophagy inhibitor and therapeutic application.

## Supplementary Material

Supplementary figures and tables.

## Figures and Tables

**Table 1:** Identification analysis of chemical constituents of* Cynomorium coccineum* in ion mode of mass spectrometry

No.	TR (min)	Name	Formula	Detected m/z	Expected m/z	M-X	Error (ppm)	Fragment ion information
1	0.469	Rhein	C15 H8 O6	284.03255	284.03209	-	1.61	283.0253, 239.0170,
2	0.472	Gallic acid	C7 H6 O5	170.02072	170.02152	-	-4.73	169.0134, 125.0239, 107.0134
3	0.505	Gentisic acid	C7 H6 O4	154.02592	154.02661	-	-4.46	153.0186, 109.02905,
4	0.959	Uridine	C9 H12 N2 O6	244.06848	244.06954	-	-4.43	243.0612, 111.0083
5	4.143	Salicylic acid	C7 H6 O3	138.03107	138.03169	-	-4.55	137.0238,109.0298,
6	5.378	Gallocatechin	C15 H14 O7	306.07271	306.07395	-	-4.07	305.0654, 137.0238, 109.0290
7	5.766	Procyanidin B2	C30H26O12	578.13984	578.14243	-	-4.46	577.13257,
8	6.236	Cianidanol	C15 H14 O6	290.07779	290.07940	-	-4.29	289.0705,245.0443
9	6.588	Syringic acid	C9 H10 O5	198.0518		-	4.56	197.0445,123.0811
10	6.819	Phloretin	C15 H14 O5	274.08302	274.08412	-	-4.02	273.07578
11	6.962	Epicatechin-3-O-gallate	C22H18O10	442.08799	442.09000	-	-4.55	441.08072, 289.0706, 245.0802, 203.0706, 169.0135, 125.0239
12	7.883	Rutin	C27H30 O16	610.15083	610.15338	-	-4.18	609.14355,301.03183, 271.02374, 255.02881
13	8.553	Taxifolin	C15 H12 O7	304.05705	304.05830	-	-4.13	303.04977,257.04449, 151.00368, 125.81577
14	9.090	Quercetin	C15 H10 O7	302.04140	302.04265	-	-4.15	301.0341,245.0430,178.99809 121.0279, 151.00307,
15	9.917	Genistein	C15 H10 O5			-		269.0074,159.0442
16	9.941	Genistein-4',7-dimethyl ether	C17 H14 O5	298.08283	298.08412	-	-4.34	297.0755, 269.0445
17	12.262	medicagenic acid	C30 H46 O6	502.32728	502.32944	-	-4.29	501.31998, 455.3148, 441.0801
18	12.693	poricoic acid A	C31 H46 O5	498.33243	498.33452	-	-4.19	497.3251, 423.2933
19	12.780	poricoic acid G	C30 H46 O5	486.33244	486.33452	-	-4.28	485.3251, 411.2522
20	13.038	Oleanolic acid	C30 H48 O3	456.3587	456.3576	+	-3.56	457.3307, 439.3199, 395.2764
21	14,638	Ergosterol peroxide	C28H44O3	428.3288	428.3290	+	-1.51	411,3258, 393.3152, 191.1066, 269.1899
22	13.57	pomolic acid	C30 H48 O4	472.35304	472.35526	-	-4.70	471.34579
23	13.67	18β-Glycyrrh-etinic acid	C30 H46 O4	470.33754	470.33961	-	-4.41	469.33026, 279.2228
24	13.590	Myristic acid	C14 H28 O2	228.20795	228.20893	-	-4.27	227.2006,194.5614
25	14.167	Betulinic acid	C30 H48 O3	456.35829	456.36035	-	-4.50	455.3510, 439.3199, 409.2348,
26	14.277	Betulin	C30 H50 O2	442.38072	442.38108	+	-0.81	411.2717, 399.1898
27	14.19	Palmitic Acid	C16 H32 O2	256.23897	256.24023	-	-4.19	255.23169
28	14.320	Oleic acid	C18 H34 O2	282.25456	282.25588	-	-4.67	281.2427
29	14.687	Stearic acid	C18 H36 O2	284.27025	284.27153	-	-3.20	283.2629

## Abbreviations

DMEM: Dulbecco's modified Eagle's medium
FBS: fetal bovine serum
PAGE: polyacrylamide gel electrophoresis

## References

[1] Meng HC, Wang S, Ying LI, Kuang YY (2013). Chemical constituents and pharmacologic actions of Cynomorium plants. Chinese Journal of Natural Medicines.

[2] Rosa A, Rescigno A, Piras A, Atzeri A, Scano P, Porcedda S (2012). Chemical composition and effect on intestinal Caco-2 cell viability and lipid profile of fixed oil from Cynomorium coccineum L. Food & Chemical Toxicology An International Journal Published for the British Industrial Biological Research Association.

[3] Sdiri M, Li X, Du WW, El-Bok S, Xie YZ, Ben-Attia M (2018). Anticancer Activity of Cynomorium coccineum.

[4] Rosa A, Nieddu M, Piras A, Atzeri A, Putzu D, Rescigno A (2015). Maltese Mushroom (Cynomorium coccineum L.) as Source of Oil with Potential Anticancer Activity. Nutrients.

[5] Maria José GA, Alessandra P, Silvia P, Bruno M, Danilo F, Carlos C (2015). Antifungal activity of extracts from Cynomorium coccineum growing wild in Sardinia island (Italy). Natural Product Research.

[6] Zucca P, Rosa A, Tuberoso C, Piras A, Rinaldi A, Sanjust E (2013). Evaluation of Antioxidant Potential of “Maltese Mushroom” (Cynomorium coccineum) by Means of Multiple Chemical and Biological Assays. Nutrients.

[7] Jing W, Yan R, Wang Y (2015). A practical strategy for chemical profiling of herbal medicines using ultra-high performance liquid chromatography coupled with hybrid triple quadrupole-linear ion trap mass spectrometry: a case study of Mori Cortex. Analytical Methods.

[8] Li X, Wu Q, Xie Y, Ding Y, Du WW, Sdiri M (2015). Ergosterol purified from medicinal mushroom Amauroderma rude inhibits cancer growth in vitro and in vivo by up-regulating multiple tumor suppressors. Oncotarget.

[9] Du WW, Fang L, Yang W, Wu N, Awan FM, Yang Z (2017). Induction of tumor apoptosis through a circular RNA enhancing Foxo3 activity. Cell death and differentiation.

[10] Du WW, Yang W, Liu E, Yang Z, Dhaliwal P, Yang BB (2016). Foxo3 circular RNA retards cell cycle progression via forming ternary complexes with p21 and CDK2. Nucleic acids research.

[11] Fang L, Du WW, Awan FM, Dong J, Yang BB (2019). The circular RNA circ-Ccnb1 dissociates Ccnb1/Cdk1 complex suppressing cell invasion and tumorigenesis. Cancer letters.

[12] Du WW, Yang W, Li X, Awan FM, Yang Z, Fang L (2018). A circular RNA circ-DNMT1 enhances breast cancer progression by activating autophagy. Oncogene.

[13] Yang Q, Du WW, Wu N, Yang W, Awan FM, Fang L (2017). A circular RNA promotes tumorigenesis by inducing c-myc nuclear translocation. Cell death and differentiation.

[14] Fang L, Du WW, Lyu J, Dong J, Zhang C, Yang W (2018). Enhanced breast cancer progression by mutant p53 is inhibited by the circular RNA circ-Ccnb1.

[15] Du WW, Yang W, Chen Y, Wu ZK, Foster FS, Yang Z (2017). Foxo3 circular RNA promotes cardiac senescence by modulating multiple factors associated with stress and senescence responses. European heart journal.

[16] Zeng Y, Du WW, Wu Y, Yang Z, Awan FM, Li X (2017). A Circular RNA Binds To and Activates AKT Phosphorylation and Nuclear Localization Reducing Apoptosis and Enhancing Cardiac Repair. Theranostics.

[17] Gong Z, Chen S, Wu Z, Jing M, Kai D, Zhou H (2018). Rapid qualitative and quantitative analyses of eighteen phenolic compounds from Lycium ruthenicum Murray by UPLC-Q-Orbitrap MS and their antioxidant activity. Food Chemistry.

[18] Ma CM, Jia SS, Sun T, Zhang YW (1993). TRITERPENES AND STEROIDAL COMPOUNDS FROM CYNOMORIUM SONGARICUM. Yao xue xue bao = Acta pharmaceutica Sinica.

[19] Zhanhu C, Zhiqin G, Jianhua M, Zhenwang W, Qianquan L, Xingyun C (2013). The genus Cynomorium in China: an ethnopharmacological and phytochemical review. Journal of Ethnopharmacology.

[20] Wang Z, Qu Y, Wang L, Zhang X, Xiao H (2016). Ultra-high performance liquid chromatography with linear ion trap-Orbitrap hybrid mass spectrometry combined with a systematic strategy based on fragment ions for the rapid separation and characterization of components in Stellera chamaejasme extracts. Journal of Separation Science.

[21] Li X Xie Y Yang BB Characterizing novel anti-oncogenic triterpenoids from ganoderma Cell cycle 20170 10.1080/15384101.2017.1315493 PMC5969549 28402700

[22] Motohiko Ukiya, Toshihiro Akihisa, †, Harukuni Tokuda, Masaya Hirano, Manabu Oshikubo, Yoshitoshi Nobukuni (2002). Inhibition of Tumor-Promoting Effects by Poricoic Acids G and H and Other Lanostane-Type Triterpenes and Cytotoxic Activity of Poricoic Acids A and G from Poria cocos. Journal of Natural Products.

[23] Bednarczyk-Cwynar B, Zaprutko L, Ruszkowski P, Hładoń B (2012). Anticancer effect of A-ring or/and C-ring modified oleanolic acid derivatives on KB, MCF-7 and HeLa cell lines. Organic & Biomolecular Chemistry.

[24] Kabeya Y, Mizushima N, Ueno T, Yamamoto A, Kirisako T, Noda T (2000). LC3, a mammalian homologue of yeast Apg8p, is localized in autophagosome membranes after processing. Embo Journal.

[25] Hua H, Yongjun D, Fangyan D, Zekun G, Jiaxue W, Xinyu S (2003). Post-translational modifications of three members of the human MAP1LC3 family and detection of a novel type of modification for MAP1LC3B. Journal of Biological Chemistry.

[26] Kabeya Y, Mizushima N, Yamamoto A, Oshitani-Okamoto S, Ohsumi Y, Yoshimori T (2004). LC3, GABARAP and GATE16 localize to autophagosomal membrane depending on form-II formation. Journal of Cell Science.

[27] Tanida I, Ueno T, Kominami E (2008). LC3 and Autophagy. Methods Mol Biol.

[28] Zhang J, Ney PA (2009). Role of BNIP3 and NIX in cell death, autophagy, and mitophagy. Cell Death & Differentiation.

[29] Jain MV, Paczulla AM, Klonisch T, Dimgba FN, Rao SB, Roberg K (2013). Interconnections between apoptotic, autophagic and necrotic pathways: implications for cancer therapy development. Journal of Cellular & Molecular Medicine.

[30] Imazu T, Shimizu S, Tagami S, Matsushima M, Nakamura Y, Miki T (1999). Bcl-2/E1B 19 kDa-interacting protein 3-like protein (Bnip3L) interacts with bcl-2/Bcl-xL and induces apoptosis by altering mitochondrial membrane permeability. Oncogene.

[31] Harborne JB, Saito N, Detoni CH (1994). Anthocyanins of Cephaelis, Cynomorium, Euterpe, Lavatera and Pinanga. Biochemical Systematics & Ecology.

[32] Nie H, Wang Y, Qin Y, Gong X (2016). Oleanolic acid induces autophagic death in human gastric cancer cells in vitro and in vivo. Cell Biology International.

[33] Kim B, Kim J, Park B (2016). Pomolic Acid Inhibits Invasion of Breast Cancer Cells Through the Suppression of CXC Chemokine Receptor Type 4 Expression. Journal of Cellular Biochemistry.

[34] Kabeya Y, Mizushima N, Ueno T, Yamamoto A, Kirisako T, Noda T (2000). LC3, a mammalian homologue of yeast Apg8p, is localized in autophagosome membranes after processing. The EMBO journal.

[35] Tsujimoto Y, Finger LR, Yunis J, Nowell PC, Croce CM (1984). Cloning of the chromosome breakpoint of neoplastic B cells with the t(14;18) chromosome translocation. Science.

[36] Schenk RL, Strasser A, Dewson G (2017). BCL-2: Long and winding path from discovery to therapeutic target. Biochemical & Biophysical Research Communications.

[37] Bellot G, Garciamedina R, Gounon P, Chiche J, Roux D, Pouysségur J (2009). Hypoxia-Induced Autophagy Is Mediated through Hypoxia-Inducible Factor Induction of BNIP3 and BNIP3L via Their BH3 Domains. European Journal of Cancer Supplements.

[38] Shigeru D, Takao K, Akitsugu Y, Hayato T, Yasuko K, Seiji K (2004). Pivotal role of the cell death factor BNIP3 in ceramide-induced autophagic cell death in malignant glioma cells. Cancer Research.

[39] Nan Z, Yali C, Ruixuan J, Erwei L, Xiuling C, Zhijun X (2011). PARP and RIP 1 are required for autophagy induced by 11'-deoxyverticillin A, which precedes caspase-dependent apoptosis. Autophagy.

[40] Sowter HM, Ratcliffe PJ, Watson P, Greenberg AH, Harris AL (2001). HIF-1-dependent regulation of hypoxic induction of the cell death factors BNIP3 and NIX in human tumors. Cancer Research.

