# Supplementary material for: Identification and characterization of chemical components in the bioactive fractions of Cynomorium coccineum that possess anticancer activity
Source: Int J Biol Sci. 2020 Jan 1;16(1):61–73. doi: 10.7150/ijbs.38475 (PMC6930376; doi:10.7150/ijbs.38475)
Supplement: Supplementary file 1 — Supplementary figures and tables. [file ijbsv16p0061s1.pdf]

Supplementary Information

Table S1: Sequences of primers used in the study

Gene	Primer sequence
Bcl-2	Forward5’-GTC GCA GAG GGG CTA CGA GTG GGA-3’
	Reverse 5’-ACCACAGGTGGCACCGGGCTGAGC-3’
Lc3(39)	Forward 5'-ATG CCG TCG GAG AAG AAC-3'
	Reverse 5'-TTA CAC TGA CAA TTTCAT CC-3'
BNIP3L(40)	Forward 5'-ATG CCG TCG GAG AAG AAC-3'
	Reverse 5'-TTA CAC TGA CAA TTTCAT CC-3'
BNIP3(40)	Forward 5’-ACCAACAGGGCTTCTGAAAC-3’
	Reverse 5’-GAGGGTGGCCGTGCGC-3’
U6	Forward 5’-GTGCTCGCTTCGGCAGCACATATAC-3’
	Reverse 5’- AAAAATATGGAACGCTTCACGAATTTG-3’

Fig S1

Cynomorium coccineum powder was soaked and extracted by 95% ethanol. The extracted solution was filtered to get supernatants and precipitates. The supernatants were concentrated (CS1). Furthermore, the precipitates were extracted with 75% ethanol. The extracted solution was filtered and concentrated to obtain a yield (CS2). The CS2 was then fractionated by ethyl acetate (CS3), water-saturated butanol (CS4), and water alone stepwise (CS5).

Fig S2

HepG2 cells were treated with CS3 (0–40 μg/mL) for 24 h. Cells lysate was prepared and analyzed by western blotting probed with anti-Bcl-xL antibody.

Fig S1

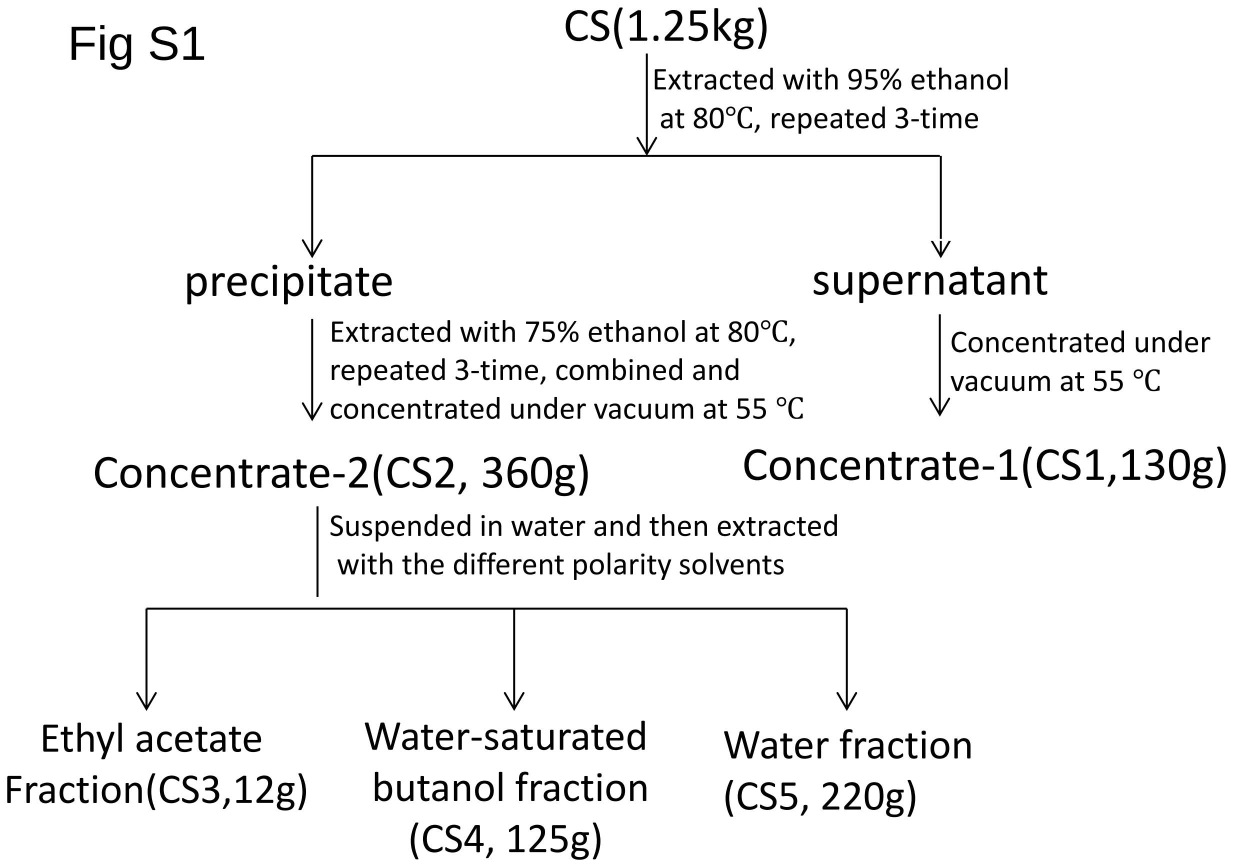
CS(1.25kg)
Extracted with 95% ethanol at 80℃, repeated 3-time
precipitate
supernatant
Extracted with 75% ethanol at 80℃, repeated 3-time, combined and concentrated under vacuum at 55 ℃
Concentrated under vacuum at 55 ℃
Concentrate-2(CS2, 360g)
Concentrate-1(CS1,130g)
Suspended in water and then extracted with the different polarity solvents
Ethyl acetate Fraction(CS3,12g)
Water-saturated butanol fraction (CS4, 125g)
Water fraction (CS5, 220g)

Fig S2

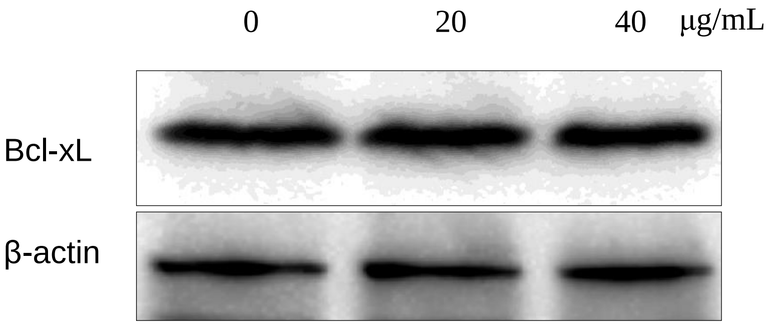
0
20
40
μg/mL
Bcl-xL
β-actin
